# Selective targeting of transforming growth factor-beta1 into TCR/CD28 signalling plasma membrane domains silences T cell activation

**DOI:** 10.1186/s12964-014-0074-6

**Published:** 2014-12-08

**Authors:** Thomas Harder, Karina Guttek, Lars Philipsen, Luca Simeoni, Burkhart Schraven, Dirk Reinhold

**Affiliations:** Institute for Molecular and Clinical Immunology, Otto-von-Guericke University Magdeburg, Leipziger Straße 44, D 39120 Magdeburg, Germany

**Keywords:** Silencing of T lymphocyte activation, TGFβ1, Plasma membrane compartmentalisation, Biologicals for treatment of transplant rejection, Allergies, Autoimmunity

## Abstract

TGFβ1 (Transforming Growth Factor-beta1) is a versatile regulator of T cell immune responses. Depending on its context in the immunological environment, TGFβ1 guides T cells toward specific activation programs including T_H_17 and regulatory T cell activities. Moreover, TGFβ signals function in immune homeostasis by directly attenuating T cell effector activities. We uncovered a novel context under which TGFβ1 stringently and reversibly silences activation responses of resting human T cells to TCR/CD28 stimulating surfaces:

Using ligand-presenting beads, TGFβ1 and TCR/CD28-activating signals were directed into defined plasma membrane domains of T cells. Selective targeting of TGFβ1 cytokine into TCR/CD28 signalling plasma membrane domains held back early response of TCR-proximal tyrosine phosphorylation and bead engulfment at activation sites. Consequently, downstream induction of proliferation and cytokine secretion were stringently attenuated. After extended incubation with TGFβ1-presenting beads, silenced T cells became receptive again to activation by renewed TCR/CD28-stimuli, indicating that the unresponsive state of T cells was reverted and did not reflect long-lasting anergy or decrease in T cell viability. These findings outline a new strategy of physically linking TGFβ1 and TCR-activating functions for the treatment of disease and pathological conditions which are caused by unwanted T cell activity.

**Electronic supplementary material:**

The online version of this article (doi:10.1186/s12964-014-0074-6) contains supplementary material, which is available to authorized users.

## Lay abstract

The human body is permanently exposed to pathogens like viruses which infect cells of the body and cause disease. In order to control pathogen attack, humans and animal species have evolved a sophisticated defence; the immune system. The immune system needs to recognise pathogens, fend them off, and memorise these pathogens in case they reappear. However, loss of control over these defence mechanisms is a cause of many serious diseases like diabetes, inflammatory bowel disease, multiple sclerosis, and allergies. In the present study we developed a set-up under which key cells of the immune system; so-called T cells, are held in a dormant state under which they do not activate their functions in immune defence. This new route of T cell-silencing promises a lead toward treatment of numerous important diseases which are caused by unwanted action of T cells like diabetes, inflammatory bowel disease, multiple sclerosis, and allergies.

## Background

Transforming growth factor-beta (TGFβ) has pleiotropic roles in steering differentiation and homeostasis of cells, tissues, and organs [1]. TGFβ1 is a versatile regulator of T cell immune responses and guides CD4+ T lymphocytes toward specific activities of the T_H_17 and Treg phenotypes [2,3]. Additionally, TGFβ signals restrain T cell activities and thereby directly function in immune homeostasis [4,5]. Functions of TGFβ1 in safeguarding a healthy balance of the immune system were highlighted by severe multiorgan inflammatory disease of TGFβ1-deficient mice [6,7]. Dysfunction of TGFβ1-mediated T cell balance is associated with autoimmune disorders and thus molecular principles of TGFβ-regulation of T cell activities are of important medical interest.

TGFβ signals are transduced by TGFβ receptors (TGFβR) I and II which catalyse serine/threonine phosphorylation of SMAD proteins. Phosphorylated SMADs cooperate with various transcription factors to activate cell-specific gene transcription programs. TGFβ receptors, cytoplasmic and nuclear factors, and chromatin structure determine context-dependent cellular outcomes of these canonical TGFβ signalling cascades. Additional non-canonical TGFβ signalling pathways exist, many of which are initiated at the platform of the plasma membrane for example by targeting PI3K/AKT pathway and Rho-GTPase signalling cascades [1].

Under physiological, resting conditions TGFβ is held in an inactive latent configuration in a complex with Latency Associated Peptide (LAP) which hinders TGFβ cytokine binding to TGFβ receptors. Latent TGFβ gets activated when active cytokine is released from TGFβ-LAP complex. Various mechanisms mediate activation of TGFβ: These include specific binding of proteins like thrombospondin to LAP, LAP proteolytic cleavage, αv integrin-binding and cytoskeletal force along membrane-bound TGFβ-LAP complex [8-10].

T cell antigen receptor (TCR), upon interaction with a cognate peptide-MHC ligand, transduces key signals which activate resting T lymphocytes to proliferate and perform immune effector functions. These signals are propagated to the T cell interior via tyrosine kinase activation and induction of signalling complexes which mediate intracellular signalling reactions and respective T cell responses [11]. A costimulatory second signal is mediated by surface protein CD28 on engagement with CD80/86 antigens on antigen-presenting cells and is required for full activation of resting T lymphocytes to proliferate and perform effector functions like secretion of cytokines [12].

TGFβ1-LAP is presented on the surface of CD4+ CD25+ T cells and was proposed to exert regulatory functions upon contact with effector T cells [13,14]. On FoxP3+ regulatory T cells (Tregs) TGFβ1-LAP is bound to trans-plasma membrane protein GARP (glycoprotein A repetitions predominant protein) [15,16]. siRNA-mediated depletion of GARP in human Tregs reduced, but did not abolish, suppressive activity of FoxP3+ Tregs *in vitro*, indicating contribution of TGFβ1 surface presentation as well as additional functions to regulatory activities of Tregs [16]. A distinct population of FoxP3+ human Tregs expresses MHC class II and was shown to specifically mediate early suppression of T cell activation directly upon cell/cell contact [17].

TCR activation and TGFβ1-signals may likewise act together at the immunological synapse between CD4+ T cells and antigen-presenting dendritic cells (DC) in order to induce Treg and T_H_17 activities. These DCs need to express matching MHC class II to activate TCR and αv integrins which present TGFβ1 to T cells [18-20].

Hence, simultaneous presentation of TGFβ1 and TCR-activating ligands on cell surfaces is a recurring motif in modulation of T cell activities. Here we used defined ligand-coated beads as artificial surfaces to present activating stimuli to resting human T cells *in vitro*: Simultaneous presentation of TGFβ1 and TCR/CD28-signals in common T cell plasma membrane signalling domains stringently and reversibly held back activation responses of T cells. These findings identified lateral plasma membrane compartmentalization of TGFβ1 signalling as cell biological context under which T cell activation responses are attenuated and suggest new strategies for treatment of disease and pathological conditions which are caused by unwanted T cell activity.

## Results and discussion

### Presentation of TGFβ1 on TCR/CD28-activating beads silences T cell activation responses

**Figure 1:**
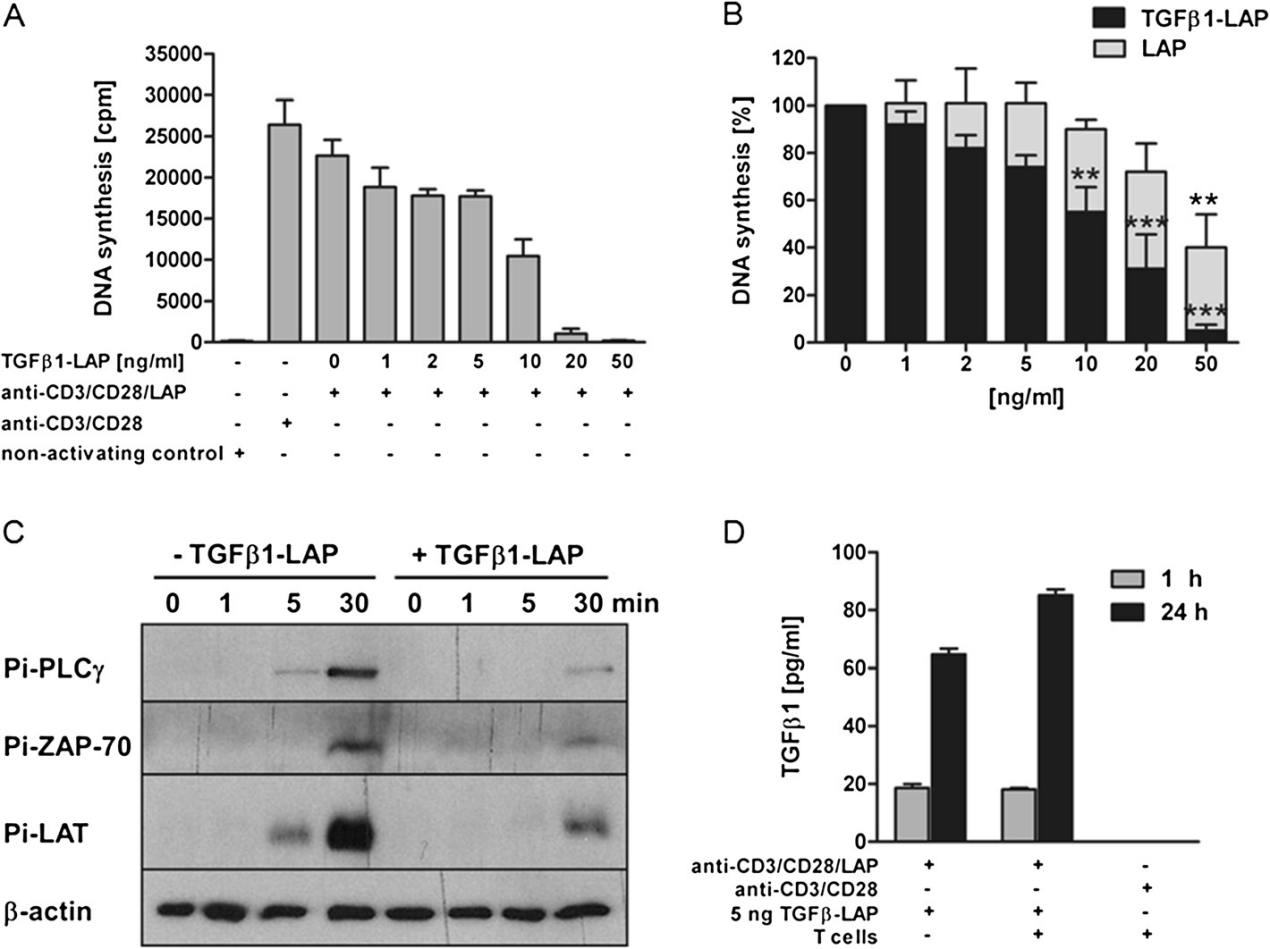
**A-D Attenuation of T cell activation response by TGFβ1-LAP-loaded on anti-CD3/CD28/LAP beads. A)** TGFβ1-LAP on anti-CD3/CD28/LAP activating beads, shown in ng/ml final concentration, holds back bead-induced proliferation of human T lymphocytes in dose-dependent manner, nearing a complete attenuation at highest doses. Proliferation was assayed by [^3^H]thymidine incorporation, depicted in counts per minute (cpm). Data present averages and SEMs of 3 independent experiments with hexuplicate cultures each. **B)** Inactive LAP and TGFβ1-LAP were loaded onto anti-CD3/CD28/LAP beads in parallel titrations. TGFβ1-LAP on activating beads recapitulated dose-dependent silencing of T cell proliferative response to TCR/CD28 activation. Loading of LAP elicited significant inhibition of proliferation only at highest doses (n = 4). **, *** depict respective significant difference of p <0.01, 0.005 to 100% proliferation response elicited by anti-CD3/CD28/LAP beads. **C)** Western blots show that bead-induced tyrosine phosphorylation of TCR-proximal signalling proteins PLCγ, ZAP-70, and LAT is attenuated by TGFβ1 presentation on anti-CD3/CD28/LAP beads. β-actin was probed as a loading control. Representative anti-phosphotyrosine blots of 3 independent experiments are shown. **D)** Gradual release of TGFβ1 cytokine by TGFβ1-LAP-loaded beads into the aqueous supernatant of ELISA wells.

Isolated resting human T lymphocytes were activated via TCR/CD28 by conjugation with protein G-coated microbeads which were coated with anti-CD3 and anti-CD28 mAbs (monoclonal antibodies) and, additionally, with mAb against the LAP subunit of latent TGFβ-LAP complex. Defined amounts of TGFβ1-LAP were loaded onto anti-CD3/CD28/LAP beads via anti-LAP antibodies. TGFβ1-LAP-loading silenced proliferative response of T cells to bead-bound anti-CD3 and anti-CD28 mAbs in strictly dose-dependent manner; first evident around 2 ng/ml final concentration and nearing completion at 50 ng/ml (Figure 1A).

We estimated contribution of steric impediment of antibody access to T cells by bead-bound TGFβ1-LAP to attenuation of T cell proliferation response. In parallel experiments TCR/CD28-activating beads were loaded with inactive LAP and with latent TGFβ1-LAP. Inactive LAP, when loaded on T cell-activating anti-CD3/CD28/LAP beads, partially inhibited proliferation at highest doses (Figure 1B) indicating that steric impediment of activating antibody access contributes to the inhibitory effect. This, however, can be discriminated from strictly dose-dependent and stringent antiproliferative activity of TGFβ1-LAP on activating beads.

We then assessed whether presentation of TGFβ1-LAP on anti-CD3/CD28/LAP beads also attenuated early response of tyrosine phosphorylation of TCR-proximal signalling proteins [21]. Western blots of whole cell lysates of TCR/CD28 activating bead-stimulated T cells were performed over a time course of 30 mins, 37°C incubation. These blots reiterated TCR triggering-induced tyrosine phosphorylation of tyrosine kinase ZAP-70, transmembrane Linker for Activation of T cells (LAT) and phospholipase C γ (PLCγ), with respective Mw at 70 kD, 36 kD and 110 kD (Figure 1C). Importantly, when TGFβ1-LAP was presented by TCR/CD28-activating beads, tyrosine phosphorylation of ZAP-70, LAT, and PLCγ was reduced. These data suggest that silencing of T cell activation responses upon TGFβ1-LAP presentation on TCR/CD28-activating beads commences with attenuation of early TCR-proximal tyrosine phosphorylation.

Holding back T cell activation response by local presentation of latent TGFβ1-LAP implied mechanisms which release active TGFβ1-cytokine toward TCR/CD28 activation domains. ELISA analysis of bead supernatants indicated spontaneous release of TGFβ1-cytokine from beads at a slow rate (Figure 1D) which may be sufficient for the T cell-silencing mechanism described here. Presence of T cells elicited an additional ~30% release of TGFβ1 at 24 h 37°C incubation. Association/dissociation kinetics of TGFβ-LAP complexes and of TGFβ with TGFβRI and II were comprehensively characterized in plasmon resonance studies [22], unravelling rates of spontaneous slow dissociation and rapid rebinding of TGFβ1 to LAP, consistent with our results. In our ELISA assays bead-bound non-complexed LAP molecules compete with TGFβ-capturing ELISA antibodies for binding free TGFβ1 cytokine. Moreover, T cellular activities partially contribute to latent TGFβ1-LAP activation. In conclusion, our ELISAs demonstrated gradual release of active TGFβ1 cytokine from beads but could not estimate quantities and rates in T cell/bead-conjugates.

### TGFβ and TCR/CD28 signals cooperate in common plasma membrane domains in order to silence T cell proliferative response

**Figure 2:**
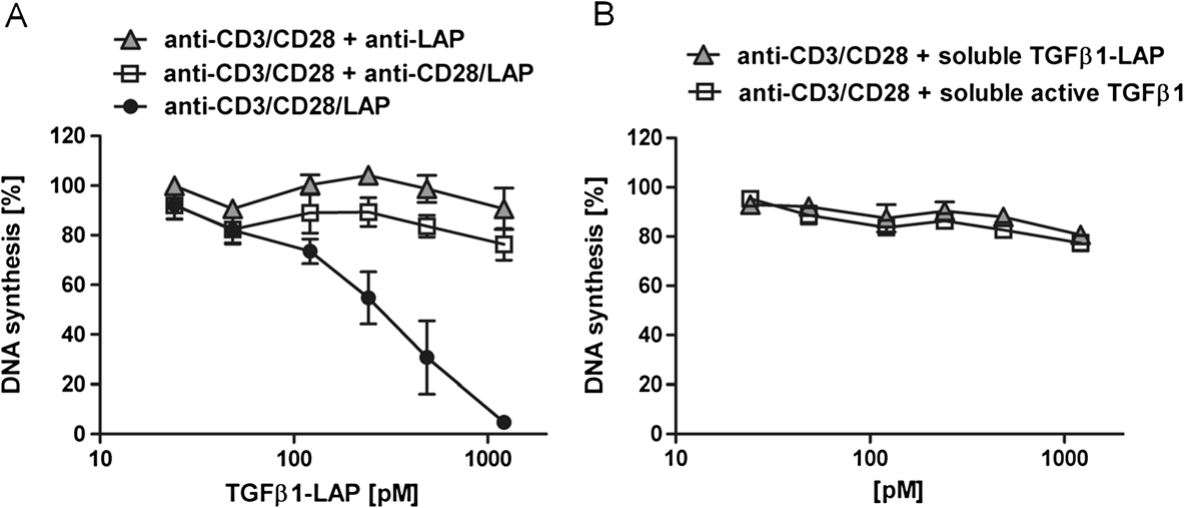
**A, B Selective targeting of TGFβ1 into TCR/CD28 activation sites attenuates T cell proliferation response. A)** TGFβ1-LAP, when presented to human T lymphocytes by anti-LAP- (grey triangles) or anti-LAP/CD28- (white squares) antibody-coated protein G beads, does not inhibit proliferative response elicited by separate anti-CD3/CD28 antibody-coated beads (n = 3). TGFβ1-LAP presented on anti-CD3/CD28/LAP beads (black circles) effectively held back proliferation (n = 4). **B)** Soluble TGFβ1, as latent TGFβ1-LAP (black circles) or active TGFβ1 cytokine (white squares), was present in equimolar amounts as TGFβ1-LAP for Figures 1A, 2A and did not effectively hold back T cell proliferation induced by activating anti-CD3/CD28 beads.

We next queried the requirement of simultaneously presenting TCR/CD28 activating stimulus and TGFβ1 activity on the same bead for holding back T-cell proliferation (Figure 2AB). This was addressed by presenting anti-CD3/CD28 stimulus and TGFβ1-LAP on separate beads (Figure 2A). TGFβ1-LAP was presented by protein G beads which were either coated with anti-LAP antibodies alone or with anti-LAP and anti-CD28 antibodies at a 1:1 ratio in order to enhance contact of anti-LAP beads with T cells. Importantly, both ways of presenting TGFβ1-LAP to T cells had little influence on proliferative response induced by anti-CD3/CD28 beads. This indicated that stringent silencing of T cell proliferative response only occurred when TCR/CD28 activating stimuli and TGFβ1-LAP were presented to T cells together on the same bead.

TGFβ1 was presented to T cells as soluble latent TGFβ1-LAP complex or as soluble active TGFβ1 cytokine and elicited little antiproliferative effect on T cells which were activated by anti-CD3/CD28 protein G beads (Figure 2B). It is particularly important to emphasise that, at this specific setting of T cell activation, soluble active TGFβ1 cytokine could not substitute for the lack of TGFβ1 presentation on T cell activating beads in blocking activation response; suggesting that TCR/CD28- and TGFβ-signals integrate and cooperate in common T cell plasma membrane domains and then provide signals which silence T cell activation response. Interestingly, MHC class II expression identifies a distinct population of human Tregs which mediates early suppression of effector T cell activation responses on direct cell/cell contact [17]. It is tempting to speculate that silencing beads mimic suppressive activities of this MHC class II + Treg population in the human system.

### Diminished proliferative response of bead-silenced T cells to TCR/CD28-activating culture plates

**Figure 3:**
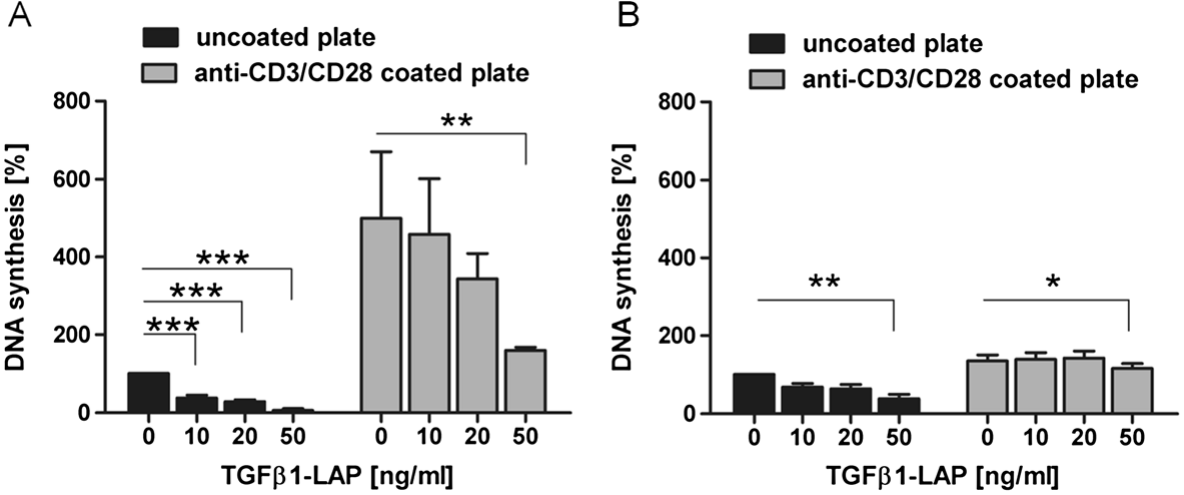
**Attenuated proliferative response of bead-silenced T cells to TCR/CD28-activating culture dishes.** T cells were incubated in round-bottom tubes with anti-CD3/CD28/LAP beads which were loaded with indicated amounts of TGFβ1-LAP. After **A)** 15 min and **B)** 24 h bead/T cell activation reactions were transferred onto tissue culture plates which were either left untreated (black bars) or were coated with anti-CD3 and anti-CD28 antibodies (grey bars) *, **, *** depict significant (p <0.05, 0.01, 0.005) attenuation of proliferation rates when T cells were preincubated with TGFβ1-LAP on activating beads. **A)** Transfer of 15 min-incubated bead/T cell conjugates to uncoated plates recapitulated dose-dependent attenuation of T cell proliferation by TGFβ1-LAP on activating beads. These T cells strongly responded to independent anti-CD3/CD28 stimulus on culture plates. This additional proliferative response was inhibited by initial TGFβ1-LAP-presentation in dose-dependent manner; down to ~31% at highest loading of TGFβ1-LAP (n = 4). **B)** After 24 h of bead/T cell preincubation T cells responded with additional proliferation to plate-bound anti-CD3/CD28 antibodies. Proliferation on anti-CD3/CD28 plates, however, was restored to nearly the same rates with and without TGFβ1-LAP preincubation on beads; down to ~85% at highest doses (n = 3).

We observed that TGFβ1 elicits potent inhibition of T cell proliferative response when presented in physical proximity of activating TCR/CD28 stimuli. We sought to characterize response of these TGFβ1-silenced T cells when activated by fresh, independent TCR/CD28 stimulus (Figure 3AB). First, T cells were silenced for 15 min at 37°C with TGFβ1-LAP on anti-CD3/CD28/LAP beads (Figure 3A). T cell/bead conjugates were then transferred to tissue culture plates which were either left untreated or were coated with anti-CD3 and anti-CD28 antibodies. After 3 days on plates T cell proliferation rates were determined. ^3^H-thymidin incorporation was used to measure T cell proliferation rates. On transfer to uncoated non-activating plates proliferation was induced by activating beads without TGFβ1-LAP loading at 15635 ± 9310 cpm SEM and was taken as 100% proliferative response value. Variation and relatively low proliferation rates may be caused by variable loss of T cell/bead contact on transfer. Dose-dependent and stringent antiproliferative action of bead-bound TGFβ1-LAP, however, was always recapitulated. Transfer of 15 min-incubated T cells/beads to anti-CD3/CD28-coated tissue culture plates strongly enhanced T cell proliferation rates. Proliferative response to plate-bound anti-CD3/CD28-activating stimulus was significantly attenuated to ~31% by TGFβ1-LAP presentation on initial activating beads. These results indicate that T cells which were silenced by anti-CD3/CD28/LAP bead-presented TGFβ1 for 15 min exhibited a reduced response to fresh TCR/CD28 activating surface.

Next, initial bead/T cell activation reactions were incubated for 24 h before transfer to tissue culture plates (Figure 3B): On transfer to uncoated plates bead-induced proliferation without TGFβ1-LAP was measured at 47881 ± 12792 cpm SEM. Again proliferation of T cells was inhibited by TGFβ1-LAP loaded on TCR/CD28-activating beads (Figure 3B). Here however, TGFβ1-mediated suppression of T cell proliferation was less pronounced than for bead/T cell conjugates after 15 min preincubation in Figure 3A. This suggested that after 24 h of preincubation silencing activity of TGFβ1-LAP-loaded TCR/CD28-activating beads was reduced, and that silenced T cells reverted to be receptive to renewed proliferative stimulus by fresh contact with bead-bound anti-CD3/CD28 antibodies on transfer. Indeed, 24 h-preincubated T cells responded to anti-CD3 and anti-CD28 on plates with little difference in proliferation rates whether the 24 h-preincubation was performed with or without TGFβ1-LAP on activating beads. In conclusion, silencing of T cells by TGFβ1-LAP-loaded beads was reverted on longer incubations and thus did not reflect a long-lasting T cell anergic state or T cell death (Additional file 1: Figure S1 documents that T cell viability is unaltered under all experimental conditions).

### A range of T cell functions is attenuated when TGFβ1 is presented on activating beads

**Figure 4:**
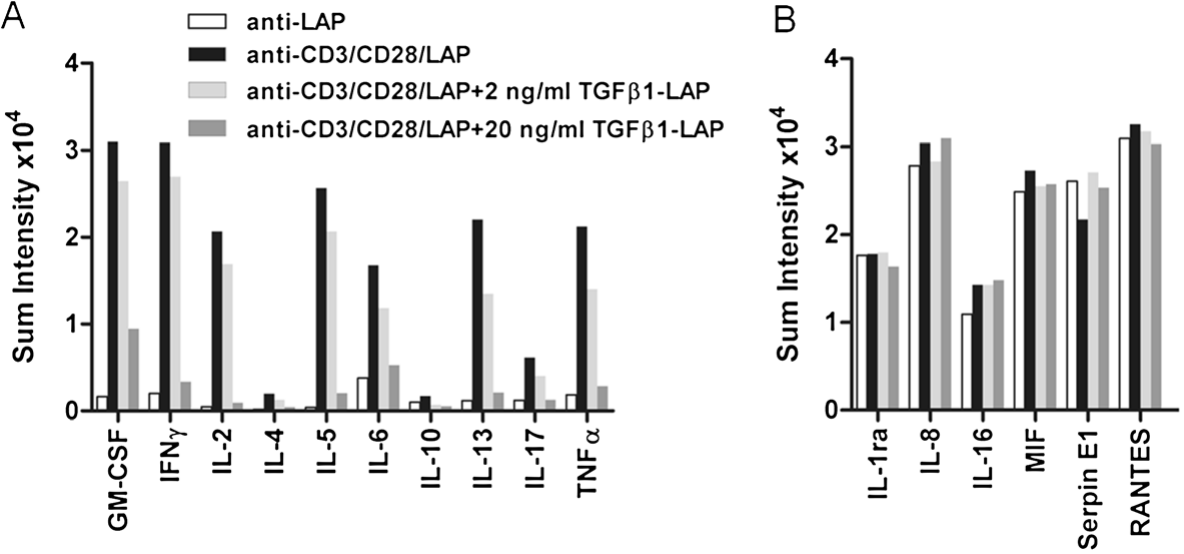
**A, B Cytokine array of human pan T cell cytokine secretion-profiles. A)** TCR/CD28 activating-antibodies on anti-CD3/CD28/LAP beads induced cytokine secretion (black bars) over non-activating control beads (white bars). Loading of TGFβ1-LAP on these TCR/CD28 activating beads suppressed activation-induced secretion of cytokines in a dose-dependent manner (grey bars). **B)** Cytokines which were secreted by resting T cells showed little induction following TCR-activation were not affected by TGFβ1 on TCR/CD28 activating beads. The cytokine array analysis was performed once and is detailed in Additional file 2: Figure S2.

A pan human T cell cytokine array was used to characterize the range of T cell effector responses which were silenced by TGFβ1-presentation on TCR/CD28-activating beads (Figure 4). As expected, activating beads alone induced secretion of cytokines IFN-γ, IL-2, IL-4, IL-10, IL-17, TNF-α. Consistent with attenuated TCR-proximal tyrosine phosphorylation and T cell proliferation, bead-bound TGFβ1-LAP held back secretion of these cytokines in dose-dependent manner to nearly baseline levels (Figure 4A). T cell effector functions which were attenuated by TGFβ1-LAP on activating beads included inflammatory responses (TNF-α), T_H_1 and T_H_2 functions (IFN-γ, IL-4), T_H_17 functions (IL-17) and regulatory T cell activities (IL-10). Other cytokines, like IL-8 and IL-16 were secreted by resting T cells and showed little induction following TCR-activation (Figure 4B). Consistently, secretion of these cytokines was not altered by TGFβ1 presentation on TCR/CD28 activating beads. Cytokine array analysis was performed once and is fully documented in Additional file 2: Figure S2.

### TGFβ1-presentation hinders spreading of T cells over TCR/CD28-activating bead surface

**Figure 5:**
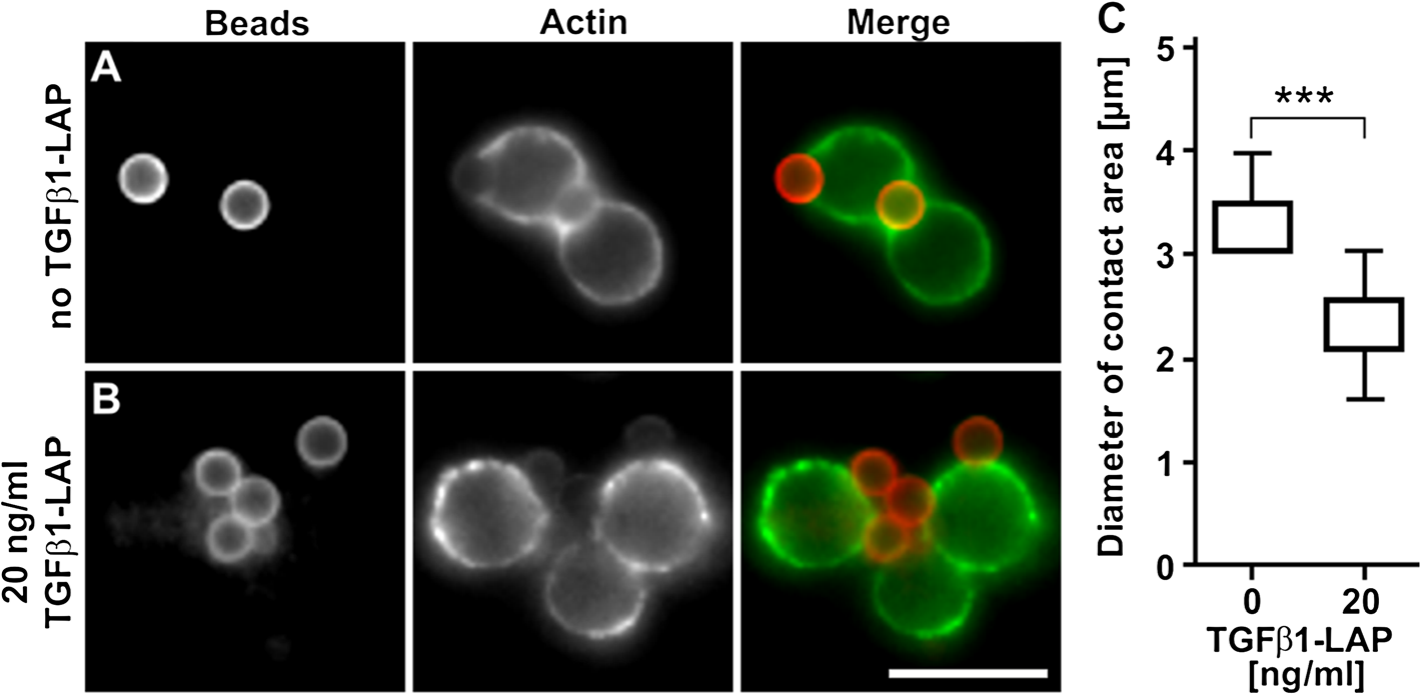
**A, B Spreading of T cells over TCR/CD28-activating bead surface.** Isolated human resting T cells were conjugated with anti-CD3/CD28/LAP beads and incubated at 37°C for 45 min and analysed by fluorescence microscopy. Outline of T cells was marked with fluorescent Alexa488 anti-actin mAb (in green in merged image, please note weak binding of Alexa488 mAb to protein G beads), protein G beads with Alexa555 IgG1 mAb (in red). **A)** Anti-CD3/CD28/LAP beads triggered spread of cells over bead surface leading to their partial or complete engulfment. Actin was depleted from the centre of bead/ T cell contact region and actin-rich lamellipodia formed at the periphery. **B)** TCR/CD28-mediated bead-engulfment by T cells and actin reorganisation was suppressed in conjugates with TGFβ1-presenting activating beads. Scale bar =10 μm. **C)** Bar graphs indicate diameter of contact area of 23 (0 ng/ml TGFβ1) or 14 (20 ng/ml TGFβ1) bead/cell conjugates in two independent experiments. Boxes mark the 25% and 75% percentiles and error bars 5% and 95% percentiles of the contact secants. *** mark significant difference of p < 0.0001 in two-tailed Mann–Whitney testing. Please note that 5% and 25% percentiles in data sets of 0 ng/ml TGFβ1 cannot be graphed separately.

Next, spreading of T cells over anti-CD3/CD28/LAP bead surfaces with and without TGFβ1-LAP loading was characterized by epifluorescence microscopy and statistical analysis (Figure 5). Bead/T cell conjugates, after 45 min incubation at 37°C, were stained with Alexa488 fluorescent anti-actin mAb to visualize the circumference of T cells, and protein G beads were additionally marked with Alexa555 mouse IgG1. Consistent with previous reports, anti-CD3/CD28/LAP antibodies alone induced T cell-spreading over the surface of TCR/CD28-activating beads leading to their engulfment (Figure 5A) and actin-containing structures formed at the edges of the beads while actin was depleted from the central region of bead-cell contact [23,24]. Importantly, loading of TGFβ1-LAP on activating beads significantly inhibited engulfment of TCR/CD28-activating beads (Figure 5B, C). Inhibition of T cell spreading over TCR-activating surfaces was reported to attenuate T cell activation response [23,25].

## Conclusions

Pleiotropic action and consequent severe side effects preclude use of active TGFβ in medical applications for the treatment of pathological conditions connected to unwanted T cell activity [26]. Our findings suggest a new strategy to circumvent these difficulties by selective targeting of TGFβ1 to TCR activation plasma membrane domains in order to specifically silence T cell responses. Bifunctional biologicals which link functions of TCR activation and TGFβRI and II activation proximally in the plasma membrane promise applications in therapy of disease associated to unbalanced, unwanted T cell activity in autoimmunity, allergy or transplant rejection.

## Methods

### Materials

Protein G-coated dynabeads were obtained from Novex Life technologies. Pan T cell isolation kit II was from Miltenyi Biotec. HIT3a anti-CD3 mAb and CD28.2 anti-CD28 mAb were from Becton Dickinson. Anti- LAP-TGFβ mAb; latent TGFβ1,; active TGFβ1 cytokine, cytokine array kit and TGFβ cytokine ELISA assay were from R&D Systems. Anti-phospho-PLCγ1 (Tyr783), anti-phospho-Zap-70 (Tyr319), and anti-phospho-LAT (Tyr191) were obtained from Cell Signaling Technology, anti-β-Actin monoclonal antibody from Sigma. HRP-conjugated donkey anti-mouse IgG or donkey anti -rabbit IgG (Dianova, Hamburg, Germany) were used as secondary antibodies. Ficoll was from Biochrome, [^3^H]thymidine from MP Biomedicals, AIM-V culture medium from Invitrogen.

### Isolation of resting human T cells

Human peripheral blood mononuclear cells (PBMC) were isolated by Ficoll gradient centrifugation of heparinized blood collected from healthy volunteers. Human T cells were further purified by non-T cell depletion using the Pan T cell isolation kit II (Miltenyi Biotec). Purity of isolated pan T cells was ≥99%. Cells were washed twice and resuspended in serum-free AIM-V culture medium (Invitrogen). The study was approved by the local ethics committee of the Medical Faculty of the Otto-von-Guericke University Magdeburg. Blood donors gave written informed consent.

### Ligand-coating of beads, ELISA analysis, and preparation of anti-CD3/CD28 antibody-bound tissue culture plates

0.6 mg of protein G dynabeads were coated with 11 μg of antibody mix; anti-CD3, anti-CD28 and anti-LAP antibodies at 1:5:5 ratios; anti-LAP, anti-CD28 antibodies at 1:1 ratios, or anti-LAP antibodies alone. After wash in 2×1 ml PBS, 0.5% BSA beads were suspended in 800 μl PBS, 0.5% BSA for microscopy, ELISAs, array analysis, and proliferation assays or in 80 μl PBS, 0.5% BSA for preparing Western blot samples. Antibody-coated beads in required minimal volumes were incubated with TGFβ1-LAP to achieve final concentrations of 0, 0.1, 0.2, 0.5, 1, 2, and 5 ng/ 4 μl of bead suspension. Anti-CD3/CD28/LAP beads for Western blot samples were loaded to 20 ng TGFβ1-LAP /4 μl suspension. Bead/TGFβ1-LAP mix was incubated o/n on a rotating wheel at 4°C, suspended in 1 ml of PBS, 0.5% BSA in ice and harvested with a magnet. Pelleted beads were then suspended in PBS, 0.5% BSA, retaining above concentrations of beads and bead-bound TGFβ1-LAP.

ELISA: 3 μg anti-CD3/CD28/LAP protein G beads were loaded with 5 ng latent TGFβ1-LAP, transferred into 100 μl AIMV medium in commercial TGFβ-ELISA wells (BD Life Technologies) and incubated for 1 h (grey bars) and 24 h (black bars) at 37°C. 10^5^ T cells were present in some wells. Plates were developed according to manufacturer’s instruction in quadruplicate wells per condition.

Plastic tissue culture plates were coated with goat anti-mouse secondary antibodies (Dianova) 1:100 in PBS 50 μl/well o/n at 4°C, washed in PBS, and then incubated for 1 h with hybridoma supernatants of anti-CD3 (Okt3) and anti-CD28 (CD28.2) monoclonal antibodies; 1:100 in PBS.

### Proliferation-, cytokine secretion-assay, viability assessment and Western blot

10^5^ T cells were incubated with 4 μl (2x10^5^) of respective ligand-coated beads in round bottom wells of 96 well microtiter plates (Nunc) with 100 μl of serum-free AIM-V medium (six wells per condition). Proliferation was assessed after 3 days by measuring [^3^H]thymidine [^3^H-TdR] incorporation. [^3^H]thymidine was added at 0.2 μCi/well for 3 h. At the end of the incubation period, cells were harvested and radioisotope incorporation was measured as index of lymphocyte proliferation using a betaplate liquid scintillation counter (MicroBeta, Wallac).

Cytokine secretion profile following 3 days of cell/bead incubation was determined from 10x100 μl AIM-V T cell supernatant per condition and was assayed using commercial cytokine array kit (R&D) according to manufacturer’s instruction. Semi-quantitative analysis was performed by determining the background-subtracted sum intensity for each dot on the membrane using Kodak D1 3.6 software. Densitometry values of spots on different membranes were normalized using the results for the positive controls.

Data were analysed, graphed, and subjected to statistical analyses using Graphpad Prism software.*, **, *** depict statistical significance p <0.05, 0.01, 0.005, respectively assessed by one way Anova testing.

Cell viability was determined in parallel cell cultures by propidium iodide (PI) staining. After 4 h, 24 h, and 72 h of incubation with respective activating beads, cells were incubated with PI/RNase staining buffer (BD Pharmingen) for 15 min and percentage of PI-excluding cells was subsequently determined by FACS according to manufacturer’s instructions.

For Western blot analyses whole cell lysates were prepared from 10^6^ T cells in 20 μl AIM-V medium. T cells were incubated with 4 μl (2×10^6^) TCR/CD28 activating beads with and without TGFβ1-LAP coating, incubated at 37°C for 0, 1, 5, 30 min, and lysed in a final volume of 100 μl 1x SDS sample buffer. Proteins, 20 μl sample per lane, were separated in 10% SDS-polyacrylamide gel and transferred to Hybond N membranes by semi-dry blotting. Specific binding was visualized by chemiluminescence (Thermo Scientific, Rockford, USA).

### Microscopy

Bead/Cell conjugates were incubated at 37°C for 45 min and adhered to poly-L-lysine coated microscopy slides. After 15 min fixation with 2% PFA cells were permeabilised for 10 min with 0.2% TX100 in PBS, washed in PBS/1% BSA for 30 min. Actin of cells was labelled with an Alexa488 conjugated anti- actin antibody (BD, 558623) and protein G beads were marked with Alexa555-labelled IgG1 mAb (Cell Signaling). Fluorescence images were recorded with a CCD camera (Apogee, KX4) mounted to a fluorescence microscope (Leica, DM IRE2) using Alexa488 (Omega Filters, XF116-2) and Alexa555 (Omega Filters, XF111-2) channel settings at 63x magnification (Leica, Oil, NA 1.40, 506206). Maximal lengths of the secants in 14 (20 ng TGFβ1-LAP) and 23 (0 ng TGFβ1-LAP) at the respective bead/cell contact zones was measured. *** mark significant inhibition (p < 0.0001) of bead engulfment by TGFβ1-LAP-loading in two tailed Mann–Whitney testing.

## Additional files

Additional file 1: Figure S1. Viability of T cells is unchanged on presentation of TGFβ1-LAP on TCR/CD28-activating beads. Bead T cell conjugates were incubated for indicated times at 37°C and percentage of viable, propidium iodide-excluding cells was determined by FACS analysis. Additional file 2: Figure S2. Documentation; Cytokine array analysis. A) Arrangement of cytokine-trapping antibodies on array, B) Chemiluminescence film exposure C) Semi-quantitative analysis of cytokine-chemiluminescence signals using software Kodak D1 3.6.
