# Supplementary figures and images for: Selective targeting of transforming growth factor-beta1 into TCR/CD28 signalling plasma membrane domains silences T cell activation
Source: Cell Commun Signal. 2014 Dec 8;12:74. doi: 10.1186/s12964-014-0074-6 (PMC4258951; doi:10.1186/s12964-014-0074-6)

## Slide 1
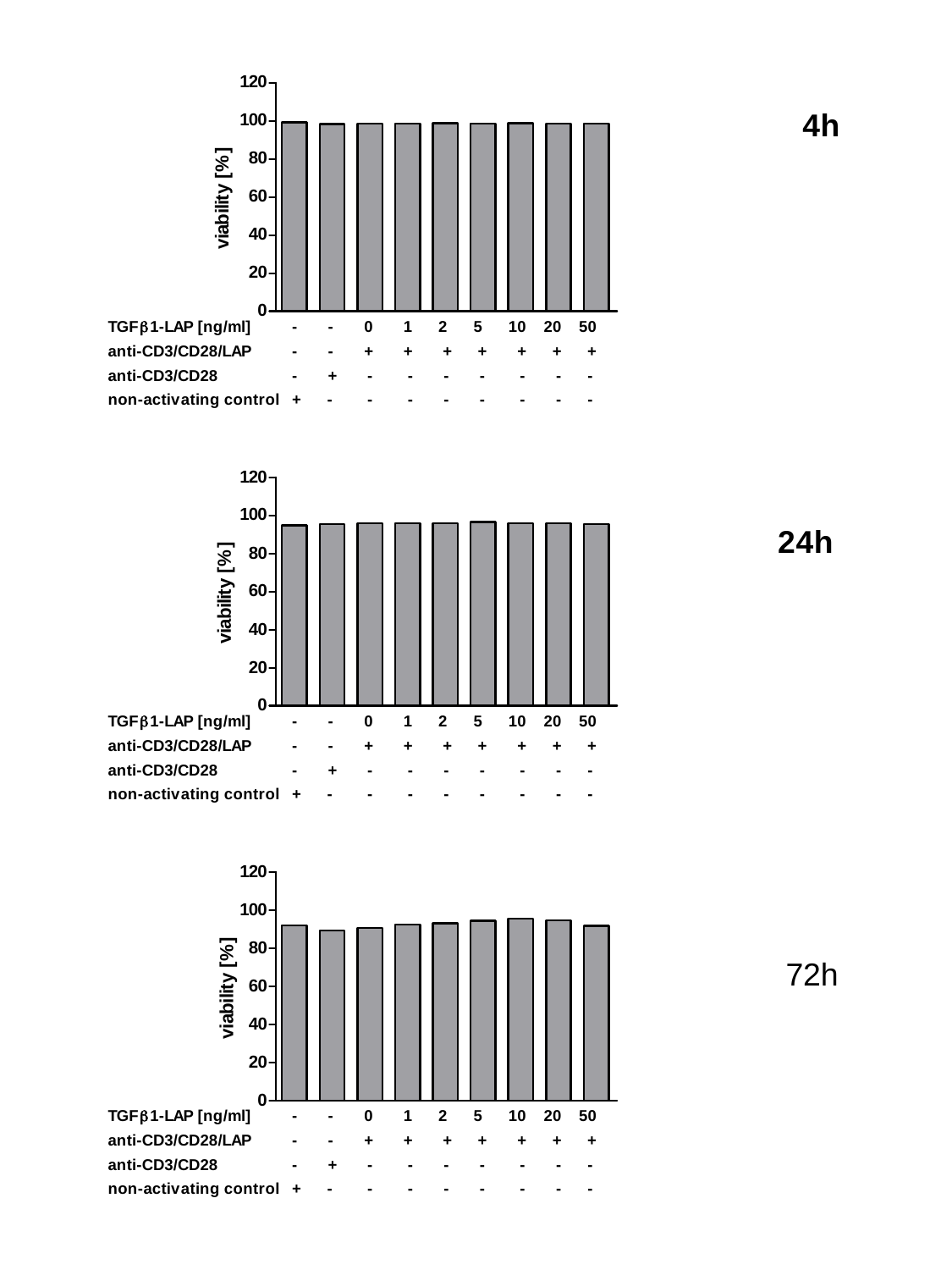

72h

Supplement: Additional file 1: Figure S1. — Viability of T cells is unchanged on presentation of TGFβ1-LAP on TCR/CD28-activating beads. Bead T cell conjugates were incubated for indicated times at 37°C and percentage of viable, propidium iodide-excluding cells was determined by FACS analysis. [file 12964_2014_74_MOESM1_ESM.ppt]

## Slide 1
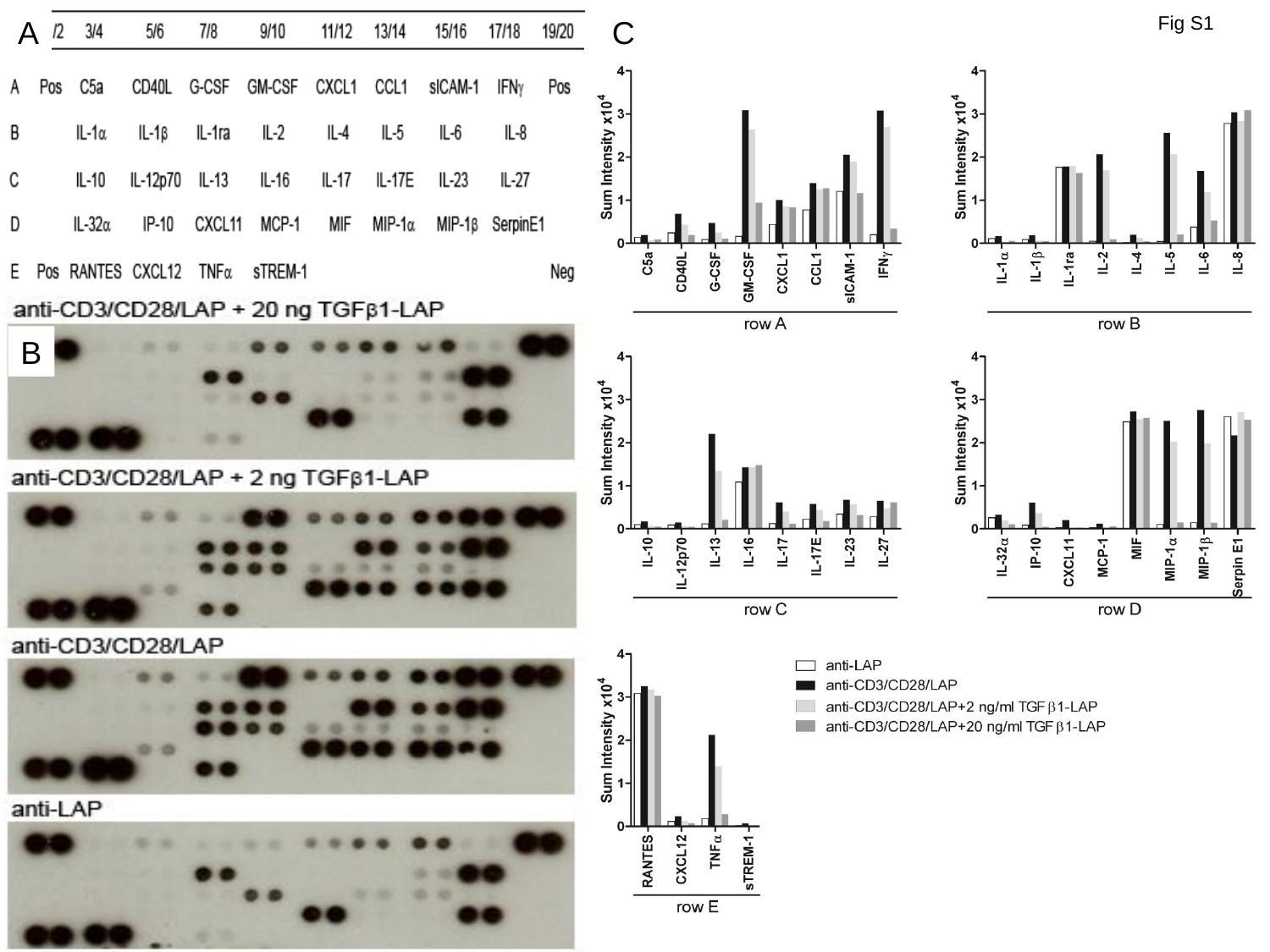

Fig S1
A
C
B

Supplement: Additional file 2: Figure S2. — Documentation; Cytokine array analysis. A) Arrangement of cytokine-trapping antibodies on array, B) Chemiluminescence film exposure C) Semi-quantitative analysis of cytokine-chemiluminescence signals using software Kodak D1 3.6. [file 12964_2014_74_MOESM2_ESM.pptx]
